# Supplementary material for: Factors of Having Difficulties Raising 3-Year-Old Children in Japan: Usefulness of Maternal and Child Health Information Accumulated by the Local Government
Source: Children (Basel). 2021 Nov 24;8(12):1084. doi: 10.3390/children8121084 (PMC8700700; doi:10.3390/children8121084)
Supplement: Supplementary file 1 [file children-08-01084-s001.zip › children-1404968-supplementary.pdf]

Table S1. Maternal and child health information

Timing of data collection	Maternal and child health information
Pregnancy notification	Notification date / Weeks of pregnancy at the time of notification (weeks) / Expected date of birth
	Age (years, pregnant women and spouse/ partner) / Height (pregnant women)
	Weight (pregnant women) / Smoker (Yes or No, pregnant women and spouse/ partner)
	Amount of cigarette per a day (pregnant women and spouse/ partner)
	Blood type (A, B, O or AB and Rh, pregnant women and spouse/ partner)
	Drink alcohol (Yes or No, pregnant women and spouse/ partner)
	Amount of alcohol (pregnant women and spouse/ partner)
	Frequency of alcohol (pregnant women and spouse/ partner)
	Number of family living together / Age of family living together
	Relationship of family living together / Health condition of family living together
	Number of pregnancies / Age of previous childbirth(years)
	Previous pregnancy abnormalities (Yes or No) / Length of previous pregnancy (weeks)
	Type of previous delivery / Child's birth weight(g) / Sex of the child/ physical condition of the child
	Morning sickness (Yes or No) / Abnormal vaginal bleeding (Yes or No)
	Lower abdominal pain (Yes or No) / Anemia (Yes or No) / Abnormal blood pressure (Yes or No)
	Disease being treated (Yes or No) / Medical history (Yes or No) / Concerns(Yes or No)
	Talk to someone (Yes or No)
Pregnant	Abnormalities in pregnancy health examination (Yes or No)
From birth to newborn	Presence of birth contact sheet (Yes or No)
	Satogaeri syussan†(Yes or No)
	Concerns(Yes or No) / Number of contacts for the Newborn visit
When visiting a newborn	Infant's gender / Weight at birth (g) / Height at birth (cm) / Chest circumference at birth (cm)
	Head circumference at birth (cm) / Delivery method / Fetal position
	Time required for delivery(minutes) / Amount of bleeding (Yes or No)
	Amount of bleeding (ml) / Abnormalities during delivery (Yes or No)
	Abnormality at birth (Yes or No) / Neonatal abnormalities (Yes or No)
	Screening test for inborn errors of metabolism (Yes or No) / Newborn hearing test (pass or refer)
	Weight at 1-month checkup (g) / Height at 1-month checkup (cm)
	Chest circumference at 1-month checkup (cm) / Head circumference at 1-month checkup(cm)
	Abnormality at the 1-month checkup (Yes or No) / Baby's age (days)
	Baby's weight at home visit (g) / Baby's weight gain(g) / Number of times to drink milk
	Nutrition (artificial, breast or mixed) / Baby's mood / Baby's complexion / Baby's musle tone
	Baby's torticollis / Limited hip joint mobility (Yes or No) / Frequency of stools
	Baby's overall judgement (no issues, observation or consultation recommendation)
	Mother's Lochia (Yes, No or not sure) / Breast milk trouble (Yes or No) / Mother's Fever (Yes or No)
	Mother's tiredness(No, strong or weak) / Mother's Sleep (Yes , No or normal)
	Mother's Appetite (Yes , No or normal) / Housework (can or can not)
	Child raising (can or can not) / Whether to return to work (Yes or No)
	Checklist for child support
	Edinburgh Postnatal Depression Questionnaire Japanese version (EPDS)
	Japanese version of Mother-to-Infant Bonding Scale (MIBS-J)
	Mother's overall judgement (no issues, observation or consultation recommendation)
Infancy	Frequency of infant health consultation attendance / Number of consultations
	Number of telephone consultations / Number of nutrition consultations
Health consultation for 4-5-month-olds	Weight at health consultation for 4-5-month-olds (g)
	Height at health consultation for 4-5-month-olds (cm)
	Chest measurement at health consultation for 4-5-month-olds (cm)
	Head measurement at health consultation for 4-5-month-olds (cm)
	Members of the family living together / Number of siblings / Birth order
	Availability of nursery school (Yes or No) / Main care provider
	Illnesses since birth (Yes or No) / Convulsions (Yes or No) / Febrile convulsion (Yes or No)
	Middle of treatment (Yes or No) / Neck stabilization (Yes or No)
	Holds a rattle or toys (Yes or No) / Any concerns about child's vision (Yes or No)
	Shows interest in a familiar voice (Yes or No) / Laugh and smile when you hold (Yes or No)
	Difficulty holding the baby (Yes or No) / Sufficient milk intake (Yes or No)
	Nutrition (artificial, breast or mixed) / Number of times to drink milk
	Receive anything other than milk (Yes or No)
	Mother's overall judgement (no issues, observation or consultation recommendation)
	Worry about baby food (Yes or No) / Enjoy raising the child (Yes, No or Not sure)
	Have time to relax and spend time with child (Yes, No or Not sure)
	Hug or call (Yes or No) / Childcare is fun (Yes, No or Not sure)
	Talk to someone about raising children (Yes or No) / Concerns(Yes or No)
18-month-old child health checkup	Main care provider during the daytime / Main care provider during the nighttime
	Illness during pregnancy (Yes or No) / Abnormality at birth (Yes or No)
	Medical history (Yes or No) / History of accident (Yes or No) / Number of febrile convulsions

	Number of convulsions / Frequent diarrhea (Yes or No) / Have had a fever (Yes or No)
	Easy to catch a cold (Yes or No) / Wheezing is difficult to stop (Yes or No)
	Prone to rash (Yes or No) / Abnormal eye movement (Yes or No)
	Middle of treatment (Yes or No) / Types of vaccinations for children
	Number of vaccinations for children / Neck stabilization (months) / Sitting up (months)
	Crawling (months) / Development consultation (free description)
	Medical consultation (free description) / Walking unsupported (months)
	Abnormal walking (Yes or No, type) / Climbs the stairs holding one hand (months)
	Medical consultation (Yes or No)
	Mimics adults' gestures (Yes or No)/ Plays with toys (Yes or No)
	Points at things to show child's interest (Yes or No) / Insufficient eye contact (Yes or No)
	Act in response to simple directions or requests (Yes or No) / Is very quiet (Yes or No)
	Point at something your child knows by looking at the picture book (Yes or No)
	When start speaking a meaningful word (months) / How many meaningful words speak
	Turns and looks when you call his/ her name? (Yes or No)
	Cries uncontrollably at night (Yes or No) / Is hyperactive (Yes or No)
	Action is at child's own pace and adult instructions are difficult to follow (Yes or No)
	Does not come to you for comfort or attention (Yes or No)
	Can your child use a spoon (Yes or No) / Wants to take off jacket (Yes or No)
	Can your child hold a cup and drink water (Yes or No)
	Talk to someone about raising children (Yes or No) / Concerns (Yes or No)
	About smoking of parents (Yes or No) / Amount of cigarette per a day
	Hope to raise children in this area (yes, if anything yes, if anything No, No)
	About father's childcare (well, sometimes, hardly or not sure)
	Taking steps to prevent drowning (Yes, No or Not applicable)
	Have time to relax and spend time with child (Yes, No or Not sure)
	Know to point to something that interests you (Yes or No) / Shake the child (Yes or No)
	Blocked my child's mouth (Yes or No) / Person brushing teeth / Know fluorine (Yes or No)
	Fluorinated (Yes or No) / Worry about teeth (Yes or No)
	What paying attention to about your child's teeth(Yes or No)
	Wake up time / Bedtime / Frequency of breakfast intake per week
	Is the meal time fixed? (Yes or No) / Breakfast time / Lunch Time / Dinner Time
	Thinking about nutritional balance (every meal, often, often not or not)
	Frequency of vegetable intake (every meal, often or not at all)
	About seasoning (light taste or not careful) / About the use of baby bottles (using or not using)
	About what child drinks most often (water or tea, milk, 100% fruit juice, soft drink,lactic acid bacteria beverage, vegetable juice, sport drink) How many times a day about snacks?
	Is the time fixed about snacks? (Yes, almost, not very, not at all or not given)
	Is the amount fixed about snacks? (Yes, almost, not very, not at all or not given)
	Favorite snacks (fruits, rice crackers, cookie, biscuits, chocolates, candies, ice creams, dairy products, rice balls, sweet breads or bubble gums)
	Does child chew food properly? (Yes, quite a lot, a little or not at all)
	Number of breakfasts with family per week / Number of dinners with family per week
	Does the child play outside? (Yes, sometimes, not much or not at all)
	Does the child enjoy the meal? (Yes, sometimes or No) / Have a food allergy? (Yes or No)
	Unbalanced diet (Yes or No) / Small appetite (Yes or No) / Over-eats (Yes or No)
	Plays with food (Yes or No) / Eating unevenness (Yes or No)
	Weight at 18 months child health checkup (g) / Height at 18 months child health checkup (cm)
	Chest circumference at 18 months child health checkup (cm)
	Head circumference at 18 months child health checkup (cm)
	Obesity judgment / Proteinuria / Urine suger / Urine occult blood (Yes or No)
	Overall judgement (no issues, observation, already medical or consultation recommendation)
	Pediatrician judgment / Dentist judgment (no issues, observation, requires inspection or need treatment)
	Dental hygienist Judgment / Nutritionist Judgment / Public health nurse (no issues, observation, follow up, already medical or consultation recommendation)
18-month-old and 3-year-old child health checkup	Transference (Yes or No) / Date entered / Person filling out / Age of parents (years)
	Health condition of parents (Yes or No) / Occupation of parents (Yes or No)
	Number of siblings / Birth order / Members of the family living together
	Maternal age (years) / Have difficulties raising your child? (always, sometimes or No)
	Too much discipline (Yes or No) / Hit emotionally (Yes or No)
	Left the infant only at home and went out (Yes or No) / Yelled emotional words (Yes or No)
	None of the abuse items apply (Yes or No)

† "Satogaeri syussan" (a traditional method for perinatal women in Japan; "Satogaeri" means returning to the original family town or house and "syussan" means delivery)
